# Populations and Health Domains Served by Direct-to-Consumer Digital Health Companies in the United States, 2011-2023: Cross-Sectional Study

**DOI:** 10.2196/78431

**Published:** 2025-11-26

**Authors:** Ashwini Nagappan, Xi Zhu, Corrina Moucheraud, Olivia S Jung

**Affiliations:** 1 Department of Health Policy and Management, Fielding School of Public Health, University of California, Los Angeles, 650 Charles Young Dr. SLos Angeles, CA, 90095, United States, 1 310 825 2594; 2 Department of Public Health Policy and Management, School of Global Public Health, New York University, New York, NY, United States

**Keywords:** digital health, direct-to-consumer, access, health equity, health services research

## Abstract

**Background:**

Direct-to-consumer (DTC) digital health companies, offering services such as on-demand prescriptions, mental health apps, fertility tracking, and at-home diagnostics, have become more common in the United States. These companies represent a shift in health care delivery by engaging consumers directly and operating largely outside of traditional health care systems. Despite their increasing presence, little is known about the populations that these companies serve, the health domains they address, and the technologies they use. Understanding these characteristics is critical for evaluating the quality of services provided, implications for health care costs, and impact on health equity.

**Objective:**

This study aimed to describe the growth and focus of DTC digital health companies in the United States from 2011 to 2023, examining their target populations, health domains, and differentiating technologies.

**Methods:**

We conducted a cross-sectional descriptive analysis using the Rock Health Digital Health Venture Funding Database, which systematically tracks US digital health companies that have received at least US $2 million in publicly disclosed funding. This database was selected because of its scope, consistency, and detailed coding of company characteristics. Of the 2652 digital health companies identified between 2011 and 2023, 478 (18.0%) were classified as exclusively pursuing a DTC model. We extracted and validated data on company characteristics, including founding year, operational status, funding levels, target populations, health domains, and technologies used. Descriptive analyses of frequencies, medians, and IQRs were conducted.

**Results:**

Between 2011 and 2023, the number of DTC digital health companies grew steadily, with the highest number founded in 2020 (59/478, 12.3%). As of 2023, 445 (93.1%) of the 478 companies remained active, and 6.9% (n=33) had ceased operations. Across all 478 companies, total venture funding ranged from US $2 million to US $570 million (median US $9.6 million, IQR US $4.0-$25.0 million). Companies focusing on rural or Medicaid populations (n=10, 2.1%) were rare and had lower median funding (median US $5.0 million, IQR US $3.5-$13.4 million). Women were the most targeted population (n=70, 14.6%), followed by children and adolescents (n=36, 7.5%), and older adults (n=25, 5.2%). Mental health was the most common health domain (n=80, 16.7%), followed by reproductive and maternal health (n=71, 14.9%). Telemedicine (n=108, 22.6%), wearables and biosensors (n=93, 19.5%), and artificial intelligence or machine learning (n=63, 13.2%) were the most frequently adopted technologies, with their use varying by population and health domain.

**Conclusions:**

As DTC digital health companies increasingly influence where and how care is delivered, systematic monitoring of their scope and characteristics is essential to evaluate whether they contribute to equitable access to care. Our findings provide a foundation for assessing whether these models are effectively addressing health needs, reaching diverse populations, and lowering health care costs.

## Introduction

Over the past decade, direct-to-consumer (DTC) digital health products and services, such as on-demand prescription services, mental health apps, fertility tracking, at-home diagnostic testing, health monitoring and tracking, and weight management platforms, have garnered increasing attention [1-3]. The growing presence of the DTC digital health market may be attributed to shifting consumer preferences and technological advances [4], as well as the COVID-19 pandemic captivating interest among entrepreneurs and investors in consumer-oriented, consumer-driven, and remote health care solutions [5,6].

**Figure 1.**
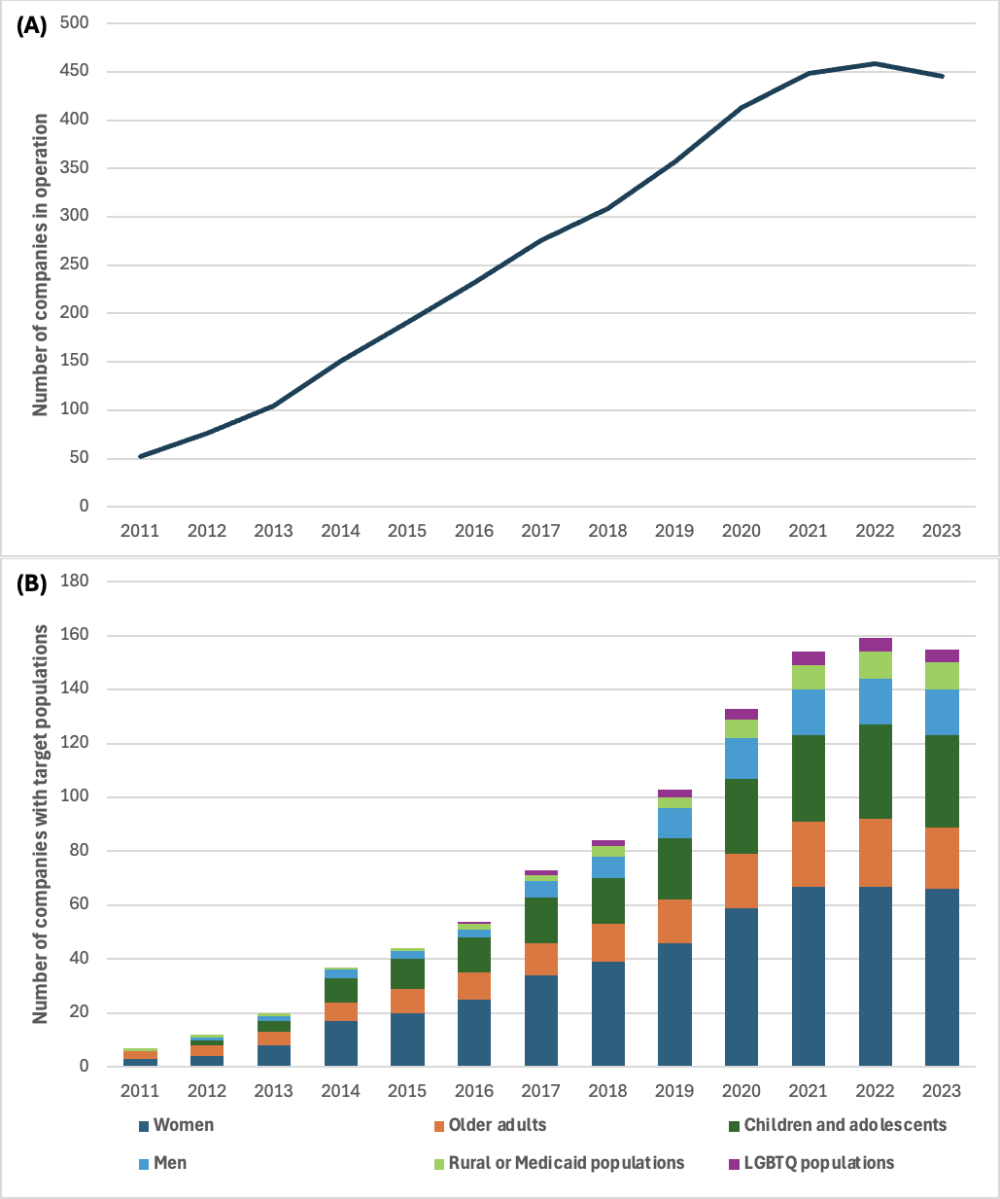
Direct-to-consumer digital health companies in operation (A) by year and (B) by target population in the United States from 2011 to 2023. LGBTQ: lesbian, gay, bisexual, transgender, and queer.

The increasing number of DTC digital health companies (Figure 1) reflects both unmet or emerging health care needs that may not be fulfilled by the traditional health care system and the ways in which companies actively create demand through direct marketing and consumer engagement [7,8]. However, there is a notable absence of scholarship understanding the populations and health domains prioritized by DTC digital health companies. A growing body of research has started to examine health care costs [9,10], consumer experiences [11-15], marketing practices [16-18], and ethical issues surrounding specific DTC digital health products and services [2,19]. Still, analyses of the DTC digital health market as a whole remain sparse, with few exceptions [1,20] and most insights coming from market reports and media coverage. Understanding who DTC digital health companies serve, what health needs they address, and how they integrate technology into care delivery is essential for assessing their implications for health care access and equity.

In this study, we used data from the Rock Health Digital Health Venture Funding Database to provide a snapshot of the growth of DTC digital health companies in the United States from 2011 to 2023, examining the populations and health domains they serve and the technologies they use. We sought to cultivate a broader understanding of which areas of care receive the most attention and resources to grasp how this modality of care delivery is being used to meet health needs.

## Methods

### Data Source

This study used the Rock Health Digital Health Venture Funding Database, maintained by Rock Health, a digital health venture capital and advisory firm. Since 2011, the database has systematically tracked digital health companies in the United States that have received over US $2 million in publicly disclosed funding. During our study period (2011-2023), the database included 2652 digital health companies. The Venture Funding Database has been used as a primary data source in peer-reviewed studies [1,20-22] and is consistently referenced for US digital health funding trends [23-25]. While other venture funding databases (eg, PitchBook, CB Insights, Crunchbase) cover broader sectors or global markets, Rock Health’s focus on digital health and its consistent application of inclusion criteria make it uniquely comprehensive for US digital health companies.

Our study relied on Rock Health’s definition of “digital health companies,” which frames them as “health companies that build and sell technologies—sometimes paired with a service, but only when the technology is, in and of itself, the service” [26]. This definition guided the scope of our sample, and it is broadly consistent with the US Food and Drug Administration’s definition of “digital health technology” as computing platforms, connectivity, software, and sensors applied to health care and related uses [27]. Data are compiled from multiple public sources, including press releases, company websites, Securities and Exchange Commission (SEC) filings, and media reports, and are continuously updated and checked for accuracy. Each company was coded based on its most recent publicly available description at the time of data validation; the database does not systematically capture historical changes in target populations, health domains, or technologies over time.

### Study Design

We conducted a cross-sectional descriptive study of digital health companies pursuing a DTC strategy. The digital health companies in this database serve several customer segments, such as consumers, payers, and employers. Given our interest in digital health companies pursuing a DTC strategy, companies were included in our sample if consumers were their exclusive customer segment. Companies that sell to payers, employers, and other segments either solely or in addition to consumers were excluded from the analytic sample. This resulted in a final sample of 478/2652 (18.0%) digital health companies in the database identified as exclusively pursuing a DTC model as of December 31, 2023.

### Variables

This unique industry dataset captured various details about the companies, including the year in which they were founded, the year in which they ceased operations (if they did), total funding received from investors, target populations (eg, women, older adults), clinical indications addressed (eg, mental health, cardiovascular disease), and differentiating technologies used (eg, telemedicine, artificial intelligence [AI] or machine learning [ML]). To ensure data accuracy and verify the companies’ operational status and the clinical indications they addressed, we conducted a thorough review of the 478 companies in our sample. First, this verification process involved cross-referencing company information with websites and public records to confirm operational status and offerings. Second, discrepancies were reviewed and reconciled by members of the research team.

About half of the companies in our sample were not tagged with a clinical indication as their product or service was not tied to a particular disease or symptom but was rather related to fitness, nutrition, or another nonclinical area. The product or service could also relate to a specific group of people with health needs (eg, older adults with fall risk). Thus, we created a new variable called “health domain” that was broader in scope than clinical indication and referred to an area of health or health care needs that a company aimed to address through its product or service. A company’s health domain denoted the clinical indication, nonclinical indication (eg, fitness), or health needs served by the company’s product or service.

Companies were not limited to a single category for target population, differentiating technology, or health domain. For example, a company could simultaneously serve older adults and lesbian, gay, bisexual, transgender, and queer (LGBTQ) individuals; use both AI or ML and wearable technologies; and address multiple health domains such as fall prevention and cardiovascular disease. Therefore, the reported frequencies reflect overlaps across categories.

### Analysis

We conducted descriptive analyses of the DTC digital health companies to examine distributions and trends by company characteristics (eg, target populations, health domains, technologies used). This included summary statistics for continuous variables, such as total venture capital funding, as well as frequency distributions for company status, health domains, differentiating technologies, and target populations. We used the median and IQR to describe the center and spread of our data as the data were not normally distributed. Multimedia Appendix 1 describes all the variables in our dataset and how they were defined. We followed the Strengthening the Reporting of Observational Studies in Epidemiology reporting guidelines [28].

### Ethical Considerations

This study involved secondary analysis of the Rock Health Digital Health Venture Funding Database, which contains company-level information collected from public sources. This study was determined to not meet the definition of human subjects research by the University of California, Los Angeles, Institutional Review Board. As the dataset contained no data on human participants, informed consent was not applicable. Privacy and confidentiality considerations were also not applicable as all records were at the company level and contained no personally identifiable or protected health information. No participants were recruited or compensated. Access to the Rock Health Digital Health Venture Funding Database was obtained through a formal data use agreement, which granted permission to use the data for academic research purposes.

## Results

The number of DTC digital health companies in the United States grew steadily between 2011 and 2023 (Figure 1), with the largest number of companies founded in 2020 (59/478, 12.3%). As of 2023, 93.1% (n=445) of the 478 companies were still in operation, with 6.9% (n=33) having ceased operations during our study period, including 42.4% (14/33) of these closures in 2023 alone. These closures were distributed across multiple health domains and target populations, without clear overrepresentation of any single category.

Figure 1 also shows that women were the most frequently targeted population, with consistent growth over the years. In 2023, 14.6% (n=70) of the 478 companies targeted women, 7.5% (n=36) targeted children and adolescents, 5.2% (n=25) targeted older adults, 3.6% (n=17) targeted men, and 2.1% (n=10) targeted rural or Medicaid populations. The number of companies focusing on men saw a slow but steady growth, particularly after 2016. Companies serving rural or Medicaid populations and LGBTQ populations began to emerge, albeit in small numbers, around 2016, signaling early steps toward more inclusive solutions in the DTC digital health space.

Table 1 shows substantial variations in funding across companies between 2011 and 2023, ranging from US $2 million to US $570 million. Companies focused on children and adolescents had the highest median funding (US $13.5 million, IQR US $6.3-$22.8 million). Companies focused on women (median US $9.4 million, IQR US $3.4-$25.0 million), men (median US $8.0 million, IQR US $6.0-$30.0 million), and older adults (median US $8.2 million, IQR US $5.2-$18.6 million) had median funding levels similar to the overall median. Companies targeting rural or Medicaid populations had consistently lower funding (median US $5.0 million, IQR US $3.5-$13.4 million; range US $2.5-$50.0 million). LGBTQ-focused companies also had a low median but exhibited much wider variation (median US $5.0 million, IQR US $3.0-$40.9 million; range US $3.0-$144.0 million), suggesting that, while most companies raised relatively modest amounts, a small number secured substantially larger deals.

**Table 1.** Venture funding across US direct-to-consumer (DTC) digital health companies by target population (2011-2023; N=478)^a^.

	Companies, n (%)	Funding (million US $), median (IQR)	Funding range (million US $)
All companies	478 (100.0)	9.6 (4.0-25.0)	2.0-570.0
Women	70 (14.6)	9.4 (3.4-25.0)	2.0-229.5
Children and adolescents	36 (7.5)	13.5 (6.3-22.8)	2.2-129.7
Older adults	25 (5.2)	8.2 (5.2-18.6)	2.0-175.0
Men	17 (3.6)	8.0 (6.0-30.0)	2.0-202.3
Rural or Medicaid populations	10 (2.1)	5.0 (3.5-13.4)	2.5-50.0
LGBTQ^b^ populations	5 (1.0)	5.0 (3.0-40.9)	3.0-144.0

a The total dataset includes 478 DTC digital health companies, with funding amounts ranging from US $2 million to US $570 million. The target population categories (eg, women or children and adolescents) represent subsets of these 478 companies, meaning that some companies served multiple populations whereas others did not have a target population. All dollar amounts are reported in nominal terms and are not adjusted for inflation.

b LGBTQ: lesbian, gay, bisexual, transgender, and queer.

DTC digital health companies targeted wide-ranging health domains (Table 2). Mental health was the most targeted health domain, with 16.7% (80/478) of all companies offering products or services for mental health conditions. This health domain was particularly prominent among companies focused on children and adolescents, with nearly 40% of them (14/36, 38.9%) addressing mental health. Reproductive and maternal health was the second most common health domain across all companies (71/478, 14.9%). Within population-focused companies, this health domain was represented in 88.6% (62/70) of the women-focused companies, 94.1% (16/17) of the men-focused companies, and 60.0% (3/5) of the LGBTQ-focused companies. The third most common health domain was fitness (68/478, 14.2%). This domain, similar to domains addressing nutrition, substance use, and oncology, was not linked to a specific target population. These findings highlight the variability in population focus across health domains, with some health issues attracting more population-specific attention, whereas others remained general in their scope.

**Table 2.** US direct-to-consumer digital health companies’ health domain focus by target population (2011-2023; N=478)^a^.

	Alln (%)	Women(n=70), n (%)	Children and adolescents(n=36), n (%)	Older adults(n=25), n (%)	Men(n=17), n (%)	Rural or Medicaid populations(n=10), n (%)	LGBTQ^b^ populations (n=5), n (%)
Mental health	80 (16.7)	12 (17.1)	14 (38.9)	1 (4)	3 (17.6)	3 (30)	1 (20)
Reproductive and maternal health	71 (14.9)	62 (88.6)	7 (19.4)	0 (0)	16 (94.1)	2 (20)	3 (60)
Fitness	68 (14.2)	1 (1.4)	0 (0)	1 (4)	0 (0)	0 (0)	0 (0)
Weight management and obesity	42 (8.8)	5 (7.1)	0 (0)	0 (0)	1 (5.9)	0 (0)	0 (0)
Primary care	40 (8.4)	11 (15.7)	3 (8.3)	1 (4)	5 (29.4)	3 (30)	0 (0)
Cardiovascular disease	23 (4.8)	4 (5.7)	1 (2.8)	0 (0)	3 (17.6)	0 (0)	0 (0)
Neurology	21 (4.4)	1 (1.4)	0 (0)	3 (12)	2 (11.8)	0 (0)	0 (0)
Diabetes	20 (4.2)	2 (2.9)	0 (0)	1 (4)	1 (5.9)	0 (0)	0 (0)
Geriatrics	18 (3.8)	0 (0)	0 (0)	15 (60)	0 (0)	0 (0)	0 (0)
Musculoskeletal and pain management	16 (3.3)	2 (2.9)	0 (0)	0 (0)	0 (0)	0 (0)	0 (0)
Gastrointestinal	15 (3.1)	4 (5.7)	1 (2.8)	0 (0)	3 (17.6)	0 (0)	0 (0)
Substance use	15 (3.1)	0 (0)	0 (0)	0 (0)	0 (0)	0 (0)	0 (0)
Developmental disorders	11 (2.3)	0 (0)	5 (13.9)	0 (0)	0 (0)	0 (0)	0 (0)
Dermatology	10 (2.1)	7 (10)	0 (0)	0 (0)	4 (23.5)	0 (0)	0 (0)
Oncology	10 (2.1)	0 (0)	0 (0)	0 (0)	0 (0)	0 (0)	0 (0)
Pediatrics	10 (2.1)	0 (0)	9 (25)	0 (0)	0 (0)	1 (10)	0 (0)
Nutrition	6 (1.3)	0 (0)	0 (0)	0 (0)	0 (0)	0 (0)	0 (0)
Allergy and immunology	5 (1)	1 (1.4)	0 (0)	0 (0)	0 (0)	0 (0)	0 (0)
Pharmacy	5 (1)	0 (0)	0 (0)	0 (0)	0 (0)	0 (0)	0 (0)
Pulmonary disorders	5 (1)	2 (2.9)	0 (0)	0 (0)	0 (0)	0 (0)	0 (0)
Audiology	4 (0.8)	0 (0)	0 (0)	0 (0)	0 (0)	0 (0)	0 (0)
Ophthalmology	4 (0.8)	0 (0)	0 (0)	0 (0)	0 (0)	0 (0)	0 (0)
Other	57 (11.9)	1 (1.4)	0 (0)	3 (12)	1 (5.9)	1 (10)	1 (20)

a The percentages in each column do not add up to 100 because some companies focused on multiple health domains. Each percentage represents the proportion of companies serving a given target population that addressed a specific health domain. Examples of “other” health domains included longevity care, wellness support, and supplements (see Multimedia Appendix 1 for more details).

b LGBTQ: lesbian, gay, bisexual, transgender, and queer.

Table 3 shows the technologies used by DTC digital health companies for each target population. Telemedicine was the most used technology, with 22.6% (108/478) of the companies using it. The second most used technology was wearables and biosensors (93/478, 19.5%), referring to devices worn on the body, such as smartwatches, fitness trackers, and other biosensors, that collect health-related data. This was followed by AI or ML technologies (63/478, 13.2%). None of the companies serving men, rural or Medicaid populations, or the LGBTQ community used wearables or AI or ML technologies. Additionally, none of the companies focused on rural or Medicaid populations used remote monitoring, which is surprising as it could be especially valuable for improving access to care and health outcomes in these communities.

The distribution of technologies across health domains shows how different technologies were applied to address various health needs (Multimedia Appendix 2). Telemedicine was primarily applied to reproductive and maternal health (30/108, 27.8%). Wearables and biosensors were primarily used in fitness (24/93, 25.8%), whereas AI, ML, or deep learning were primarily applied to mental health (13/63, 20.6%), fitness (13/63, 20.6%), and gastrointestinal conditions (7/63, 11.1%), while remote monitoring was most often used in mental health (5/28, 17.9%) and geriatrics (5/28, 17.9%).

**Table 3.** Differentiating technologies by target population of US direct-to-consumer digital health companies (2011-2023; N=478)^a^.

	Alln (%)	Women (n=70), n (%)	Children and adolescents(n=36), n (%)	Older adults(n=25), n (%)	Men (n=17), n (%)	Rural or Medicaid populations (n=10), n (%)	LGBTQ^b^ populations (n=5), n (%)
Telemedicine	108 (22.6)	26 (37.1)	17 (47.2)	6 (24)	11 (64.7)	4 (40)	2 (40)
Wearables and biosensors	93 (19.5)	6 (8.6)	5 (13.9)	6 (24)	0 (0)	0 (0)	0 (0)
AI^c^, ML^d^, or deep learning	63 (13.2)	4 (5.7)	4 (11.1)	5 (20)	0 (0)	0 (0)	0 (0)
Remote monitoring	28 (5.9)	6 (8.6)	1 (2.8)	6 (24)	1 (5.9)	0 (0)	0 (0)
Genomics	27 (5.6)	5 (7.1)	1 (2.8)	0 (0)	0 (0)	0 (0)	0 (0)
Non–medical device hardware	26 (5.4)	2 (2.9)	3 (8.3)	0 (0)	0 (0)	0 (0)	0 (0)
Other	25 (5.2)	2 (2.9)	1 (2.8)	0 (0)	1 (5.9)	0 (0)	0 (0)
Digital medical devices	13 (2.7)	5 (7.1)	0 (0)	0 (0)	0 (0)	0 (0)	0 (0)
AR^e^ and VR^f^	6 (1.3)	1 (1.4)	0 (0)	0 (0)	0 (0)	0 (0)	0 (0)
IoT^g^	5 (1)	1 (1.4)	1 (2.8)	0 (0)	0 (0)	0 (0)	0 (0)
Robotics	4 (0.8)	0 (0)	1 (2.8)	1 (4)	0 (0)	0 (0)	0 (0)
Blockchain	2 (0.4)	0 (0)	0 (0)	0 (0)	0 (0)	0 (0)	0 (0)

a The percentages in each column do not add up to 100 because some companies used multiple technologies. Each percentage represents the proportion of companies serving a given target population that used a specific technology.

b LGBTQ: lesbian, gay, bisexual, transgender, and queer.

c AI: artificial intelligence.

d ML: machine learning.

e AR: augmented reality.

f VR: virtual reality.

g IoT: Internet of Things.

## Discussion

### Summary and Significance of the Findings

Using digital health funding data, we found that the number of DTC digital health companies grew steadily between 2011 and 2023. The number of companies targeting specific populations also increased, especially those focusing on women’s health. In contrast, companies serving low-income, rural populations received considerably lower average funding, perhaps due to barriers such as limited broadband access [29] or lower out-of-pocket spending capacity. Throughout the study period, 6.9% (33/478) of the companies ceased operations, with nearly half (14/33, 42.4%) of those closures occurring in 2023. This clustering in 2023 may point to post-pandemic market corrections in digital health funding or shifts in consumer demand. Taken together, these findings illustrate how industry dynamics (eg, funding flows, consumer demand, company survival) shape the availability of DTC digital health and suggest areas where future policy attention could play a role in shaping equitable access, such as improving broadband coverage in rural and other underserved areas.

The prominence of mental health products and services among companies focused on children and adolescents indicates a responsiveness to the present-day youth mental health crisis [30]. The range of health domains addressed among DTC digital health companies revealed variation in focus, likely driven by the specific needs and demands of each population segment as well as market considerations such as total addressable market, payment models, and the perceived likelihood of attracting investment. While DTC digital health companies are interested in satisfying children’s and young adults’ unmet needs, this raises new questions about the requisite level of parental or guardian involvement in these services [31]. Specific policies may be needed to define the appropriate level of parent or guardian involvement to ensure access with safeguards to protect youth.

Nearly one-fifth of DTC digital health companies (93/478, 19.5%) used wearables and biosensors, and 13.2% (63/478) applied AI or ML, which are technologies that may support real-time monitoring and personalization. However, the collection and sharing of consumer-generated health data also raise privacy concerns, particularly in areas such as reproductive health where data misuse could lead to discrimination or legal consequences [32]. Our findings underscore the importance of monitoring how DTC digital health companies address consumer data sharing concerns [33] and whether they proactively address gaps in privacy protections without waiting for consumer health data regulations to catch up.

Future research could extend this work by examining how the strategies of specific kinds of DTC digital health companies evolve over time, as well as comparisons between companies that exclusively pursue a DTC strategy (ie, companies in our sample) and those that also serve other customer segments such as payers and employers. In addition, assessing the relationship between company characteristics and their success or survival would provide greater insights into the factors that influence which models endure and which companies cease operations. Further, additional work could assess what proportion of the DTC digital health market is represented by venture-backed start-ups versus established incumbents, particularly as this balance is shifting with increasing entry by large firms [34]; understanding these dynamics will be important for evaluating implications for competition, regulation, and equitable access.

### Comparison to Prior Work

Existing scholarship on DTC digital health has focused on particular kinds of offerings rather than on the sector as a whole. For example, studies on DTC telemedicine platforms have examined issues such as prescribing practices, continuity of care, and regulatory oversight [35]. Similarly, research on mental health apps and fertility-tracking tools has raised concerns about data privacy and effectiveness [36]. While these studies provide important insights into specific domains, less attention has been paid to systematically characterizing the broader DTC digital health landscape across populations, health domains, and differentiating technologies.

Our study differs in scope. Rather than analyzing a single type of offering, we provide a descriptive overview of the DTC digital health sector at large, drawing attention to who companies serve, which health domains they target, and what technologies they used. While our findings echo themes raised in previous domain-specific work (eg, the growth of mental health tools, concerns about equity), they also extend the conversation by situating these developments within the larger structure of the DTC digital health sector. In this way, this paper contributes empirical grounding for ongoing debates about the implications of DTC health for access, equity, and consumer protection.

### Limitations

This study had the following limitations. First, the companies listed in the database were restricted to those that have publicly disclosed investment deals of at least US $2 million and meet Rock Health’s definition of digital health. As a result, the data did not capture major technology companies (eg, Amazon, Apple) [37,38] or pharmaceutical companies (eg, Eli Lilly and Company, Pfizer) [39] that have ventured into DTC health and may underrepresent smaller seed-stage or bootstrapped firms that are part of the DTC digital health ecosystem. Second, as our analysis was limited to companies focusing exclusively on selling to consumers, our findings do not allow for comparisons with companies that offer insurance coverage. This distinction has equity implications as out-of-pocket costs may be a substantial barrier for underserved populations and influence the extent to which these groups can access DTC digital health services. Third, our dataset extended only through 2023. As additional years of data become available, future research could examine whether the trends we identified persist or shift and directly compare post-2023 developments with our findings.

### Conclusions

The DTC digital health market, now over a decade old, has reached an inflection point for assessing the needs it is fulfilling and who it is serving. Our findings provide a descriptive account of the well-established areas of DTC digital health. As individuals continue to receive care in DTC digital health settings outside of traditional health systems, it will be increasingly important to evaluate whether these offerings indeed improve outcomes and lower costs.

## Supplementary material

10.2196/78431 Multimedia Appendix 1 Study variables.

10.2196/78431 Multimedia Appendix 2 Differentiating technologies across health domains of direct-to-consumer digital health companies.

10.2196/78431 Checklist 1 STROBE checklist.

## Abbreviations

AI: artificial intelligence
DTC: direct-to-consumer
LGBTQ: lesbian, gay, bisexual, transgender, and queer
ML: machine learning
