# Supplementary material for: Populations and Health Domains Served by Direct-to-Consumer Digital Health Companies in the United States, 2011-2023: Cross-Sectional Study
Source: JMIR Form Res. 2025 Nov 26;9:e78431. doi: 10.2196/78431 (PMC12661595; doi:10.2196/78431)
Supplement: Multimedia Appendix 1 [file formative-v9-e78431-s001.docx]

**Appendix 1. Study variables**

| **Category** | **Description** | **Type** | **Operationalization** |
| --- | --- | --- | --- |
| General company details | Company | String | Company name |
|  | Founded | Numerical | Year founded |
| Investment-related details | Total funding ($M) | Numerical | Total amount of funding received in millions |
| Company product/service focus | Populations | Binary (for each) | 1 = Yes  0 = No |
|  | Differentiating technologies | Binary (for each) | 1 = Yes  0 = No |
|  | Health domains | Binary (for each) | 1 = Yes  0 = No |

1. **Population =** Primary populations that the company seeks to address or serve, respectively (if any)
   1. **Older adults**
   2. **Women**
   3. **Men**
   4. **Children and adolescents**
   5. **Underserved**
   6. **LGBTQ**
2. **Differentiating technology =** Aspect of the technology that sets the company apart; usually top-of-mind in the market; subject to change based on new technologies introduced (if any)
   1. **AI/ML/deep learning =** The use of data, algorithms and machine learning techniques (e.g., designed with the ability to learn without explicit programming) to identify the likelihood of future outcomes based on historical data and /or perform tasks that normally require human intelligence.
   2. **Wearables and biosensors =** Wearable or accessory devices (not necessarily worn) that detect specific biometrics and are intended for consumers to track themselves
   3. **Genomics and sequencing =** Hardware and software technologies that sequence, assemble, call variants, and otherwise analyze sequencing data (e.g., sequencing on a chip with data aggregation). Usually includes a data aggregation or marketplace aspects.
   4. **Telemedicine =** Technologies that enable the delivery of healthcare services (synchronous or asynchronous) from a person (not a chat-bot, automated symptom checker, etc.) when the service provider is in a different physical location from the service recipient.
   5. **Remote monitoring =** Technologies that enable the tracking and monitoring (of one person by another/others) of information when a person is not in the presence of a caregiver or provider. Requires that the information is being transmitted to another person (not self-monitoring). Enables caregiving in lower cost site of care. Also enables non-medical monitoring (typically by family/caregivers in a non-medical setting).
   6. **Augmented and virtual reality =** Technology that superimposes a computer-generated image on a user's view of the real world, thus providing a composite view or simulates an artificial environment that can be interacted with in a seemingly real or physical way by a person.
   7. **IoT =** Connected sensors that measure the physical environment (not biometrics), creating a network of "things".
   8. **Non-medical device hardware =** Connected equipment or hardware designated for professional or at-home use, that does not require FDA approval.
   9. **Robotics =** Use of robots to deliver healthcare services
   10. **Digital medical device =** Hardware, supplemented with digital capabilities, designed to diagnose, prevent, treat, mitigate, monitor or cure a disease or condition(s). Data aggregation component. Digital medical devices require FDA approval.
   11. **Blockchain =** Use of an open, distributed ledger that can record transactions between peers efficiently and in a secured, verifiable, and permanent way
3. **Health domain =** Health domain refers to a specific area of healthcare that the company seeks to address or serve, with products or services that address medical or wellness needs.
   1. **Allergy/immunology**
      1. Companies focused on the diagnosis, treatment, and management of allergies and immunological conditions, offering products and services like food allergen sensors, personalized allergy medications, and at-home diagnostic tests
   2. **Audiology**
      1. Companies providing products and services related to hearing health, including personal sound amplifiers, AI-driven audio frequency adjustment devices, and wireless hearing improvement devices
   3. **Cardiovascular disease**
      1. Companies focused on cardiovascular health, offering products like heart rate monitoring smartwatches, virtual clinics for cardiometabolic chronic diseases, and health tracker phone cases.
   4. **Dermatology**
      1. Companies providing telemedicine and online platforms for dermatological care, including virtual consultations and prescription medication delivered to door
   5. **Developmental disorders**
      1. Companies offering services for developmental disorders such as ADHD and autism, including telehealth visits, prescription medication delivery, and personalized therapy platforms
   6. **Diabetes**
      1. Companies addressing diabetes care, offering services like on-demand care platforms, smart caps for insulin pens, and mobile apps for diabetes management
   7. **Fitness**
      1. Companies promoting physical fitness through personalized workout plans, fitness tracking devices, and fitness or personal trainer apps
   8. **Gastrointestinal**
      1. Companies focused on gastrointestinal health, providing services like digestive health diagnostics, probiotics for gut health, and personalized nutrition platforms for GI issues
   9. **Mental health**
      1. Companies addressing mental health through telehealth services, AI chatbots for mental health, sleep improvement, and virtual therapy sessions
   10. **Musculoskeletal / pain management**
       1. Companies addressing musculoskeletal health or pain management solutions, offering products like digital therapeutics and wearable technology for injury prevention
   11. **Neurology**
       1. Companies addressing neurological health (e.g., conditions such as stroke, dementia, Alzheimer's, MS, and traumatic brain injury, migraine), offering services like telemedicine for neurological conditions, devices for stroke and seizure detection, and virtual reality-based therapeutics
   12. **Nutrition**
       1. Companies that address nutritional health, such as apps that simplify nutrition labels, personalized nutrition advice based on urine, and nutritional coaching without specifying weight management
   13. **Geriatrics**
       1. Companies focused on addressing the needs of older adults, such as fall detection, assistive robots, and caregiver support
   14. **Oncology**
       1. Companies focused on cancer care, providing diagnostic and treatment services, telemedicine platforms, and care coordination
   15. **Ophthalmology**
       1. Companies offering eye health services, including vision correction devices, telemedicine for eye care, and AI-based diagnostic tools
   16. **Pediatrics**
       1. Companies that address pediatric needs, such as general pediatric health, child companion robots, smartphone connected baby monitors, and baby breathing video monitors
   17. **Pharmacy**
       1. Companies that are online pharmacies; note: this is different from companies that offer prescription services
   18. **Primary care**
       1. Companies offering comprehensive primary care services through telemedicine, AI-based health management platforms, and mobile health apps
   19. **Pulmonary disorder**
       1. Companies addressing pulmonary disorders, providing telehealth services and health tracking devices
   20. **Reproductive and maternal health**
       1. Companies focused on reproductive and maternal health, offering services like hormone-based wellness apps, personalized birth control solutions, and telemedicine for women's health; also includes male and female fertility testing, menopausal care, and maternal health
   21. **Substance use**
       1. Companies that offer therapy for substance use disorder
   22. **Weight management and obesity**
       1. Companies addressing weight management and obesity through personalized nutrition platforms, diabetes care services, wellness optimization apps, or offering GLP-1s
   23. **Other**
       1. Companies offering diverse health-related services that do not fit into the specific categories listed above, including emergency response connected health systems, longevity supplements, payment management apps, health benefits administration, healthy habit apps, personalized supplements, wellness trackers, and platforms that provide retirement benefit information
