# Supplementary material for: Populations and Health Domains Served by Direct-to-Consumer Digital Health Companies in the United States, 2011-2023: Cross-Sectional Study
Source: JMIR Form Res. 2025 Nov 26;9:e78431. doi: 10.2196/78431 (PMC12661595; doi:10.2196/78431)
Supplement: Multimedia Appendix 2 [file formative-v9-e78431-s002.docx]

**Appendix 2. Differentiating technologies across health domains of direct-to-consumer digital health companies.**

|  | Total sample (n=478) | Tele-medicine (n=108) | Wearables and biosensors (n=93) | AI/ML/  Deep learning (n=63) | Remote monitoring (n=28) | Genomics (n=27) | Nonmedical device hardware (n=26) | Other (n=25) | Digital medical device (n=13) | AR/VR (n=6) | IoT (n=5) | Robotics (n=4) | Block-chain (n=2) |
| --- | --- | --- | --- | --- | --- | --- | --- | --- | --- | --- | --- | --- | --- |
| Mental health | 16.7% | 25.0% | 12.9% | 20.6% | 17.9% | 0.0% | 11.5% | 12.0% | 7.7% | 50.0% | 0.0% | 0.0% | 0.0% |
| Reproductive  and maternal health | 14.9% | 27.8% | 5.4% | 4.8% | 10.7% | 18.5% | 7.7% | 12.0% | 30.8% | 16.7% | 20.0% | 0.0% | 0.0% |
| Fitness | 14.2% | 0.9% | 25.8% | 20.6% | 10.7% | 3.7% | 46.2% | 8.0% | 0.0% | 33.3% | 20.0% | 25.0% | 0.0% |
| Other | 11.9% | 1.9% | 4.3% | 9.5% | 3.6% | 33.3% | 3.8% | 28.0% | 0.0% | 0.0% | 20.0% | 0.0% | 100.0% |
| Weight management and obesity | 8.8% | 10.2% | 14.0% | 3.2% | 3.6% | 18.5% | 3.8% | 12.0% | 0.0% | 0.0% | 0.0% | 0.0% | 0.0% |
| Primary care | 8.4% | 18.5% | 1.1% | 6.3% | 7.1% | 0.0% | 3.8% | 4.0% | 7.7% | 0.0% | 0.0% | 0.0% | 0.0% |
| Cardiovascular disease | 4.8% | 5.6% | 11.8% | 1.6% | 7.1% | 7.4% | 0.0% | 0.0% | 7.7% | 0.0% | 20.0% | 0.0% | 0.0% |
| Neurology | 4.4% | 2.8% | 8.6% | 7.9% | 10.7% | 7.4% | 3.8% | 4.0% | 0.0% | 0.0% | 0.0% | 0.0% | 0.0% |
| Diabetes | 4.2% | 5.6% | 8.6% | 3.2% | 14.3% | 7.4% | 0.0% | 0.0% | 15.4% | 0.0% | 0.0% | 0.0% | 0.0% |
| Geriatrics | 3.8% | 2.8% | 5.4% | 3.2% | 17.9% | 0.0% | 0.0% | 0.0% | 0.0% | 0.0% | 0.0% | 25.0% | 0.0% |
| Musculoskeletal/  pain management | 3.3% | 2.8% | 2.2% | 4.8% | 3.6% | 0.0% | 3.8% | 8.0% | 7.7% | 16.7% | 0.0% | 25.0% | 0.0% |
| Gastrointestinal | 3.1% | 2.8% | 1.1% | 11.1% | 0.0% | 14.8% | 0.0% | 0.0% | 0.0% | 0.0% | 0.0% | 0.0% | 0.0% |
| Substance use | 3.1% | 9.3% | 1.1% | 1.6% | 3.6% | 0.0% | 0.0% | 8.0% | 0.0% | 16.7% | 0.0% | 0.0% | 0.0% |
| Developmental disorders | 2.3% | 9.3% | 1.1% | 3.2% | 0.0% | 0.0% | 0.0% | 0.0% | 0.0% | 0.0% | 0.0% | 0.0% | 0.0% |
| Dermatology | 2.1% | 7.4% | 0.0% | 0.0% | 0.0% | 0.0% | 0.0% | 0.0% | 0.0% | 0.0% | 0.0% | 0.0% | 0.0% |
| Oncology | 2.1% | 0.0% | 1.1% | 1.6% | 0.0% | 7.4% | 0.0% | 8.0% | 0.0% | 0.0% | 0.0% | 0.0% | 0.0% |
| Pediatrics | 2.1% | 2.8% | 3.2% | 0.0% | 0.0% | 0.0% | 7.7% | 0.0% | 0.0% | 0.0% | 20.0% | 25.0% | 0.0% |
| Nutrition | 1.3% | 0.0% | 0.0% | 0.0% | 0.0% | 3.7% | 0.0% | 0.0% | 0.0% | 0.0% | 0.0% | 0.0% | 0.0% |
| Allergy/  immunology | 1.0% | 0.9% | 1.1% | 0.0% | 0.0% | 0.0% | 3.8% | 4.0% | 0.0% | 0.0% | 0.0% | 0.0% | 0.0% |
| Pharmacy | 1.0% | 0.9% | 0.0% | 0.0% | 0.0% | 0.0% | 0.0% | 0.0% | 0.0% | 0.0% | 0.0% | 0.0% | 0.0% |
| Pulmonary disorder | 1.0% | 2.8% | 2.2% | 1.6% | 0.0% | 0.0% | 0.0% | 0.0% | 0.0% | 0.0% | 0.0% | 0.0% | 0.0% |
| Audiology | 0.8% | 0.9% | 0.0% | 1.6% | 0.0% | 0.0% | 0.0% | 0.0% | 23.1% | 0.0% | 0.0% | 0.0% | 0.0% |
| Ophthalmology | 0.8% | 0.0% | 0.0% | 0.0% | 0.0% | 0.0% | 3.8% | 0.0% | 0.0% | 0.0% | 0.0% | 0.0% | 0.0% |
